# Amplified cross-linking efficiency of self-assembled monolayers through targeted dissociative electron attachment for the production of carbon nanomembranes

**DOI:** 10.3762/bjnano.8.256

**Published:** 2017-11-30

**Authors:** Sascha Koch, Christopher D Kaiser, Paul Penner, Michael Barclay, Lena Frommeyer, Daniel Emmrich, Patrick Stohmann, Tarek Abu-Husein, Andreas Terfort, D Howard Fairbrother, Oddur Ingólfsson, Armin Gölzhäuser

**Affiliations:** 1 Physics of Supramolecular Systems and Surfaces, Bielefeld University, 33613 Bielefeld, Germany; 2 Department of Chemistry, Johns Hopkins University, Baltimore, Maryland 21218, USA; 3 Department of Chemistry, Institute of Inorganic and Analytical Chemistry, Goethe-University, 60438 Frankfurt, Germany,; 4 Department of Chemistry and Science Institute, University of Iceland, Dunhagi 3, 107 Reykjavik, Iceland

**Keywords:** 2D materials, carbon nanomembrane, dissociative electron attachment, dissociative ionization, helium ion microscopy, self-assembled monolayers, X-ray photoelectron spectroscopy

## Abstract

The determination of the negative ion yield of 2′-chloro-1,1′-biphenyl (2-Cl-BP), 2′-bromo-1,1′-biphenyl (2-Br-BP) and 2′-iodo-1,1′-biphenyl (2-I-BP) upon dissociative electron attachment (DEA) at an electron energy of 0 eV revealed cross section values that were more than ten times higher for iodide loss from 2-I-BP than for the other halogenides from the respective biphenyls (BPs). Comparison with dissociative ionization mass spectra shows that the ratio of the efficiency of electron impact ionization induced fragmentation of 2-I-BP, 2-Br-BP, and 2-Cl-BP amounts to approximately 1:0.7:0.6. Inspired by these results, self-assembled monolayers (SAMs) of the respective biphenyl-4-thiols, 2-Cl-BPT, 2-Br-BPT, 2-I-BPT as well as BPT, were grown on a Au(111) substrate and exposed to 50 eV electrons. The effect of electron irradiation was investigated by X-ray photoelectron spectroscopy (XPS), to determine whether the high relative DEA cross section for iodide loss from 2-I-BPT as compared to 2-Br-BP and 2-Cl-BP is reflected in the cross-linking efficiency of SAMs made from these materials. Such sensitization could reduce the electron dose needed for the cross-linking process and may thus lead to a significantly faster conversion of the respective SAMs into carbon nanomembranes (CNMs) without the need for an increased current density. XPS data support the notation that DEA sensitization may be used to achieve more efficient electron-induced cross-linking of SAMs, revealing more than ten times faster cross-linking of 2-I-BPT SAMs compared to those made from the other halogenated biphenyls or from native BPT at the same current density. Furthermore, the transfer of a freestanding membrane onto a TEM grid and the subsequent investigation by helium ion microscopy (HIM) verified the existence of a mechanically stable CNM created from 2-I-BPT after exposure to an electron dose as low as 1.8 mC/cm^2^. In contrast, SAMs made from BPT, 2-Cl-BPT and 2-Br-BPT did not form stable CNMs after a significantly higher electron dose of 9 mC/cm^2^.

## Introduction

Carbon nanomembranes (CNMs) are two-dimensional molecular sheets with a thickness of one to a few nanometers, high mechanical strength, and high thermal stability [1–3]. Depending on the fabrication method, the choice of precursor molecules and functionalization type, such membranes have great potential for a wide variety of applications [2]. In recent years, protocols and procedures have been developed to produce functional CNMs by electron-induced cross-linking of specific aromatic self-assembled monolayers (SAMs) [4–7] and for their subsequent release from the substrate, multilayer stacking, and conversion to conductive carbon layers [8–9].

The cross-linking is typically achieved through irradiation with electrons in the energy range of 50–100 eV [10–12], but has also been realized through UV irradiation [13], ion irradiation in a helium ion microscope [14], and through high-energy electrons in the keV energy range [15]. In this context, the cross-linking is attributed to partial fragmentation of the monomers also induced by secondary electrons that are unavoidably produced through the interaction of high-energy radiation with condensed matter (photoelectrons in the case of UV irradiation) [13–14,16].

In typical aromatic monomers of SAMs, such as biphenyls, the cross-linking is primarily attributed to electron-induced C–H bond cleavage in the monomers, but also C–C cleavage [17]. In turn, the reactive radical site, generated by such bond cleavage, leads to cross-linking to neighboring monomers within the SAMs, eventually converting them into a laterally cross-linked monomolecular film (i.e., a nanomembrane). The cross-linking may be induced directly by the primary electrons, where electron exposure is used in the cross-linking step. However, as mentioned above, backscattered and secondary electrons may also play a considerable role [18].

Typically, the energy distribution of such secondary electron peaks well below 10 eV is significant at threshold (i.e., close to 0 eV), and has a high energy tail, the extension of which depends on the energy of the primary irradiation and the nature of the substrate (see, e.g., [18–20] and references therein). For both silver and copper at a primary electron energy of 30 eV, the secondary electron yield peaks below 1 eV with a full-width half-maximum (FWHM) of about 3.4 and 5.6 eV, respectively. This is considered to be a significant contribution at threshold and a tail at higher energy [21]. The situation may be expected to be similar as that of gold [22]. In the energy range from about 0–100 eV, electron-induced bond rupture may proceed through four distinctly different initiating processes: dissociative ionization (DI), neutral or dipolar dissociation upon electronic excitation (ND and DD, respectively) or through dissociative electron attachment (DEA) (see, e.g., [23–27] and literature therein).

While DI, DD and ND are nonresonant processes with thresholds above the first electronic excitation energy of the respective molecules (ND and DD) or above their ionization energy (DI), DEA is a resonant and very bond selective process leading to the production of a negative ion fragment and a neutral, radical counterpart. The initial step is the formation of a transient negative ion (TNI) through vertical transition from the ground state neutral to the respective anionic state, strained within the initial neutral geometry. The TNI is thus bound to relax through re-emission of the electron (autodetachment) or through dissociation. The cross section for the formation of the TNI at very low incident energies follows an *E*^−1/2^ energy dependency and can be substantial at or close to 0 eV [28–29]. Dissociative ionization, on the other hand, is a more statistical process with an onset slightly above the ionization limit of the respective molecules, and a maximum in the range between 40–100 eV, after which the cross sections slowly taper off as the energy transfer in the electron–molecule collision becomes less efficient.

The bond selectivity in DEA and the fact that the cross sections for this process may be significant at 0 eV electron incident energy opens up the attractive possibility to use this process to purposely enhance the cross-linking efficiency and explore the potential of site selectivity in the cross-linking step as a tool to control the properties, and ultimately, the functionality of the produced nanomembranes. However, for single bond rupture in DEA, the prerequisite at 0 eV incident electron energy (where the attachment cross sections are highest) is that the electron affinity (EA(X)) of the charge-retaining fragment (X) exceeds the respective bond dissociation energy (BDE(M−X));

[1]
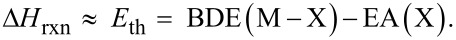


Here Δ*H*_rxn_ is the reaction enthalpy for the bond rupture, which is approximately equal to the respective threshold energy (*E*_th_). For the higher halogens Cl, Br and I, the BDE(C6H5−X) in the respective halo-benzenes decreases significantly from X = Cl (4.14 eV), to Br (3.49 eV) to I (2.82eV) [30]. The EA of these three halogens, on the other hand, is comparable (i.e., 3.61, 3.36 and 3.06 eV for Cl, Br and I, respectively) [31]. Correspondingly, the threshold for the respective DEA processes such as

[2]
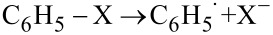


are 0.53, 0.13 and −0.24 eV for X = Cl, Br and I, respectively. For comparison, the electron affinity of hydrogen is about 0.75 eV and that of the phenyl radical is about 1.1 eV [31]. The BDE(C_6_H_5_−H) is about 4.5 eV [30], making the cleavage of a C_6_H_5_–H bond endothermic by more than 3 eV.

For the corresponding halogenated biphenyls, we expect the DEA cross section for chloro-biphenyl to be low, as this channel is not accessible where the attachment cross section is highest, that is, near 0 eV. The DEA cross section for the bromo analogue should be somewhat higher as this channel is accessible at lower energies. Finally, for the iodo-biphenyls, where the DEA channel is exothermic, we expect significant cross sections at 0 eV. Similarly, from the DEA threshold energy, we expect C–H cleavage through DEA to be present but inefficient. In fact, Houplin et al. [32] recently showed that for terphenyl-thiol (TPT) SAMs, which should behave similar to the native BPTs, DEA is an insignificant process.

The comparison of the cross-linking efficiency of SAMs made from these halogenated monomers is thus well-suited to put the hypotheses to the test that predetermined, high cross section DEA sites may be purposely used to enhance the cross-linking efficiency and eventually may offer a viable route for site-selective cross-linking. The former is desirable to speed up the large-scale production of CNMs. The latter may open up new avenues in tailoring the properties and functionality of nanomembranes.

## Results and Discussion

**Figure 1:**
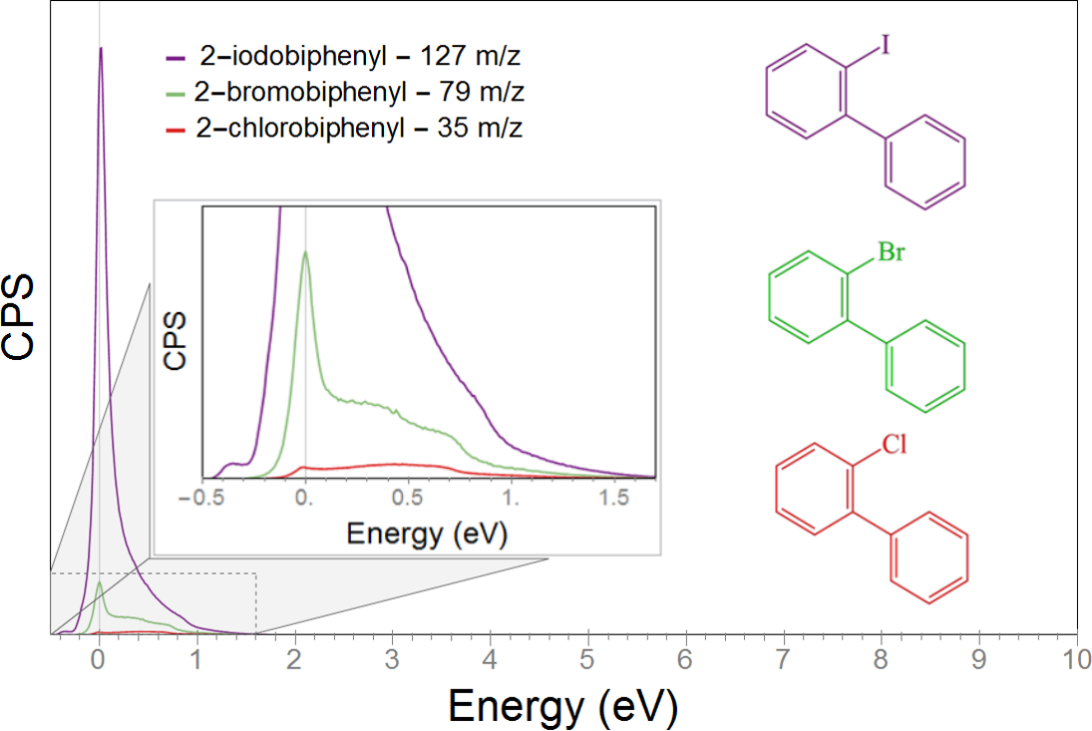
Negative halogen ion yield curves for dissociative electron attachment to 2′-chloro-1,1′-biphenyl (red), 2′-bromo-1,1′-biphenyl (green) and 2′-iodo-1,1′-biphenyl (violet) in the incident electron energy range from 0 to 10 eV. The region from 0 to about 1.5 eV is expanded to allow better comparison of the 2′-chloro-1,1′-biphenyl and 2′-bromo-1,1′-biphenyl ion yield. The respective molecular structures are shown at the top of the figure.

Figure 1 shows the energy dependence of the Cl^−^, Br^−^ and I^−^ ion yield from 2-chlorobiphenyl (2-Cl-BP), 2-bromobiphenyl (2-Br-BP) and 2-iodobiphenyl (2-I-BP), respectively, through DEA in the energy range from 0–10 eV. In DEA, the halogen loss is the only observed fragmentation channel and the formation of the halogen ions is largely confined to a fairly narrow contribution at about 0 eV. Importantly, the relative cross section for the I^−^ formation is about ten times larger than the relative cross section for the Br^−^ formation and more than 200 times larger than that for the Cl^−^ formation. As mentioned above, the electron attachment cross section at very low electron energies is proportional to *E*^−1/2^ [28–29] and the attachment cross section is thus highest (and may be very significant) at the energy threshold, that is, close to 0 eV. However, for the attachment process to lead to dissociation at these energies, the formation of the respective fragment ion must be exothermic. This is the case for the formation of I^−^ from 2-I-BP, while the formation of Cl^−^ and Br^−^ through DEA to 2-Cl-BP and 2-Br-BP, respectively, is endothermic. The fact that we still observe the formation of Cl^−^ and Br^−^ from the respective biphenyls at 0 eV (Figure 1), however, can be traced back to the internal energy distribution in these molecules at the experimental temperature, that is, the formation of Cl^−^ and Br^−^ below their thermochemical threshold is attributed to the high energy tail of the respective Maxwell–Boltzmann distribution of internal energy. The higher efficiency of the Br^−^ formation as compared to the Cl^−^ formation thus reflects the ratio of molecules with an internal energy of >0.13 as compared to >0.53 eV, rather than the actual attachment cross section.

Correspondingly, if the targeted generation of a radical site on the biphenyl moiety through DEA increases the cross-linking efficiency, it can be expected to be most apparent for 2-I-BP. The effect for 2-Cl-BP and 2-Br-BP, on the other hand, should be considerably less and only induced through transitions from the high-energy tail of their respective internal energy distributions. In a control experiment, DEA to 2-bromobiphenyl-4-thiol was also studied. The effect of the 4-thiol group was found to be insignificant.

For comparison, we have recorded positive ion mass spectra at 70 eV (not shown here), and we find that these agree well with those available in the NIST database [31]. From the current spectra, we derive an efficiency ratio of about 1:0.7:0.65 for all DI channels observed for 2-I-BP, 2-Br-BP and 2-Cl-BP, respectively. These ratios are calculated from the integral intensities of all fragments formed from the respective BPs divided by the integral intensity of the respective parent ions. These numbers show that the degree of dissociation following electron impact ionization is similar for the differently halogenated compounds. This is to be compared to an efficiency ratio of about 1:0.1:0.0025 for DEA of 2-I-BP, 2-Br-BP and 2-Cl-BP, respectively. Though these numbers cannot be considered quantitatively, they clearly show that the difference between the DEA cross sections for these compounds is significant, while that is not the case for DI.

To put the hypothesis to the test that targeted generation of a radical site by DEA may be used to increase the cross-linking efficiency, self-assembled monolayers (SAM) of biphenyl-thiols (BPTs) and halogenated biphenyl-thiols (2-Cl-BPT, 2-Br-BPT and 2-I-BPT) were prepared on an Au(111) substrate from solution (see Experimental section). Subsequently, these were irradiated by electrons at an electron energy of 50 eV. Cross-linking of the molecular layers and the formation of mechanically stable carbon nanomembranes (CNMs) was then monitored through X-ray photoelectron spectroscopy (XPS) during the irradiation process. This enables the transition of the SAMs into CNMs to be observed and allows the determination of the required dose for the respective CNM formation also by directly testing the mechanical strength and microscope images of the cross-linked SAMs.

**Figure 2:**
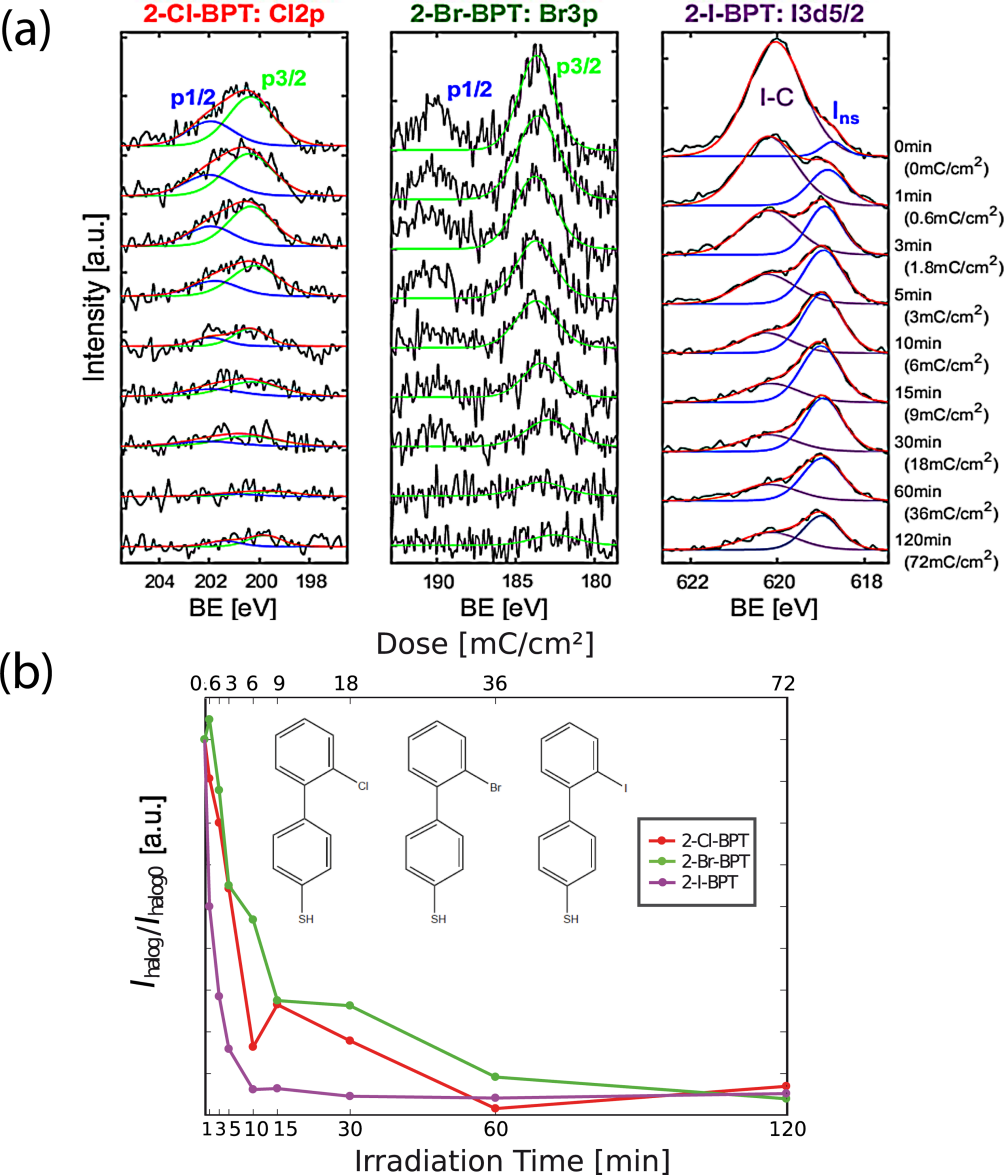
XP spectra of the Cl 2p, Br 3p doublets and the I 3d_5/2_ peak regions of SAMs made of 2-Cl-BPT 2-Br-BPT and 2-I-BPT after 0 to 120 min of electron irradiation respectively, where 1 min corresponds to an electron dose of 0.6 mC/cm^2^. (a) Raw and fitted spectra of the Cl 2p, Br 3p doublets (blue fit: p_1/2_; green fit: p_3/2_) and the I 3d_5/2_ peak of the I 3d doublet peaks of the three halogenated BPT molecules, as a function of electron dose. After electron irradiation, besides the iodine–carbon signal (I–C), a new iodine species becomes more dominant (*I*_nS_). (b) Evolution of halogen peak area *I*_halog_ for Cl (red), Br (green) and I (purple) normalized to the non-irradiated halogen peak area *I*_halog0_ due to increasing irradiation dose.

Figure 2a shows the XP spectra of the respective halogen atoms of 2-Cl-BPT, 2-Br-BPT and 2-I-BPT, recorded for nine different stages of irradiation, covering the interval from 0 to 120 min. The electron dose per minute during these experiments was 0.6 mC/cm^2^. The chlorine 2p doublet for 2-Cl-BPT is seen at 200 eV with an energy separation of 1.6 eV [33] and the bromine 3p doublet for 2-Br-BPT at 183.6 eV with an energy separation of 6.5 eV [34]. The chlorine signal is fitted by its specific doublet Cl 2p_1/2_ + Cl 2p_3/2_, while for bromine, only the Br 3p_3/2_ is used to quantify the signal of interest. It is clear from Figure 2 that the XPS intensity of the respective halogens decreases for all three molecules upon electron irradiation. For 2-I-BPT (right plot), a strong noticeable decrease in the intensity at the specific binding energy (BE) of the I 3d_5/2_ peak (I–C) at 620 eV can be observed, even after 1 min (0.6 mC/cm^2^). For the two other halogenated SAMs, the decrease of the halogen intensity is, however, rather moderate. Interestingly, in case of the iodine spectra 2-I-BPT after the decrease of the I 3d_5/2_ peak, a new peak of an iodine species (*I*_nS_) evolves at a binding energy of 619 eV. Presumably, this peak can be attributed to free iodine caused by the electron interaction forming an iodine–gold bond. A similar observation was made by Hirayama et al. while investigating dissociating iodoalkanes on a gold substrate [35].

In Figure 2b, the peak areas of the halogen peaks relative to the respective pristine SAMs are tracked and plotted versus the irradiation time and dose for 2-Cl-BPT (red curve), 2-Br-BPT (green curve) and 2-I-BPT (purple curve). All three curves show a strong exponential decrease with increasing electron irradiation. This is consistent with the expected cleaving of the halogen–carbon bonds and a subsequent detachment of the halogen atoms. This decrease in the normalized intensity, *I*_halog_/*I*_halog0_, is similar for Br 3p and Cl 2p, whereas the I 3d_5/2_ peak, exposed to the same current density, decays substantially faster. For a reduction in the normalized intensity by 50%, which corresponds to *I*_halog_/*I*_halog0_ = 0.5, in the case of bromine and chlorine, an irradiation time of 5 to 10 minutes (3–6 mC/cm^2^) is needed. For iodine, the same reduction is already reached after 1 to 3 minutes (0.6–1.8 mC/cm^2^). The intensity reduction vs irradiation dose for chlorine and bromine is rather similar, which is consistent with the fact that both of these processes are endothermic in DEA and the intensity at 0 eV, apparent in Figure 1, must thus be attributed to the high energy tail of the internal energy distribution of the respective biphenyls at the current experimental temperature.

Comparing the relative electron dose dependence of the dehalogenation process in the SAMs (Figure 2) and the relative cross sections for this process in the gas phase (Figure 1) shows that the relative difference between 2-Cl-BPT and 2-Br-BPT is less clear in the SAMs. This is to be expected due to the different conditions in the condensed phase as compared to the gas phase, for example, different temperatures and additional energy dissipation channels introduced at the surface as compared to single collision conditions in the gas phase. Furthermore, the gas phase studies shown in Figure 1 show only the halogen loss through DEA. The XPS data on the other hand show the total dehalogenation, independent of the underlying process, that is, DI, ND or DEA. Hence, if DI additionally contributes similarly to the dehalogenation of all three compounds, which is obvious from the comparable intensities of the [M–X]^+^ signals in their mass spectra [31], the difference in the DEA efficiency of this process between the three compounds will be less apparent in the XPS data. In the current SAMs, the molecules are strongly bound to the Au(111) substrate via their thiolate anchor groups and are densely packed. This introduces a non-negligible substrate and next neighbor interaction, which in turn may influence their sensitivity towards electron irradiation as compared to gas-phase molecules under single collision conditions. However, though qualitative in nature, our experiments show that the electron-induced dehalogenation process is substantially more efficient in the 2-I-BPT SAMs than the 2-Cl-BPT and 2-Br-BPT SAMs. It is reasonable to assume that the radical sites generated in the DEA process substantially stimulate the cross-linking process and that the very effective iodine loss in the DEA process should thus be reflected in the cross-linking efficiency.

The decrease of the intensity maximum of the thiol sulfur S 2p_3/2_ peak at a binding energy (BE) of 162 eV and a formation and increase of a new sulfur species with a S 2p_3/2_ peak at BE = 163.5 eV due to radiation-induced formation of new sulfur species, such as disulfides and/or thioethers [2]. This may be regarded as an indirect indicator for the transition of SAMs into CNMs and for the self-termination of the cross-linking, as already shown in previous work [16,36]. A straightforward method for the unambiguous verification of cross-linking consists of the transfer of a cross-linked SAM onto a TEM grid in order to demonstrate its mechanical strength and permit its imaging using, for instance, helium ion microscopy (HIM) [1,8].

However, since the latter procedure has to be conducted for each individual exposure time it is significantly more labor intensive. Nevertheless, for reasons of reliability, here we have applied both these approaches to follow the cross-linking of the respective halogenated biphenyls as a function of the irradiation time and thus the electron dose.

**Figure 3:**
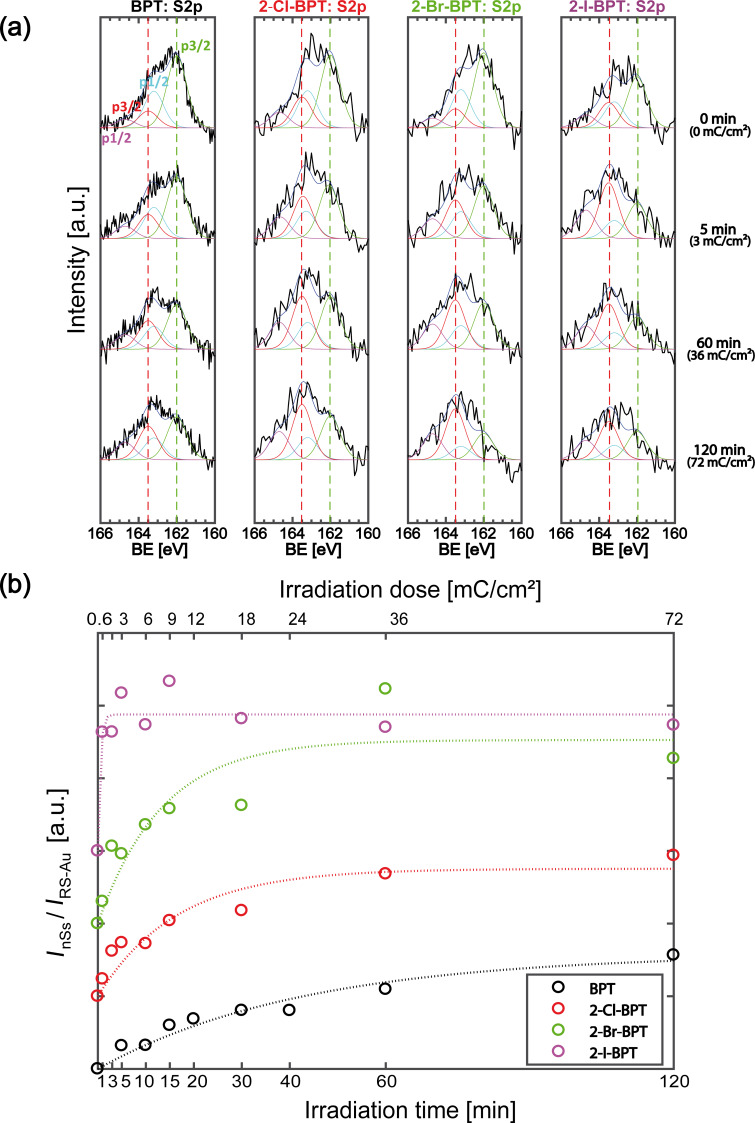
(a) Raw XP spectra of the S 2p region of SAMs made from BPT, 2-Cl-BPT 2-Br-BPT and 2-I-BPT before and after electron irradiation between 0 and 120 min (electron dose: 0–72 mC/cm^2^) equivalent to Figure 1. Fits are also shown using two S 2p_3/2_/S 2p_1/2_ doublets with an energy separation of 1.2 eV [37]; one doublet has a S 2p_3/2_ peak at 162 eV (green), and a S 2p_1/2_ at 163.2 eV (blue), and corresponds to sulfur atoms in the native SAM (RS–Au); the second doublet has a S 2p_3/2_ peak at 163.5 eV (red), and a S 2p_1/2_ at 164.7 eV (purple), and corresponds to new sulfur species (nSs) produced as a result of electron irradiation. (b) Plot of the intensity ratios *I*_nSs_ (162 eV)/*I*_RS-Au_ (163.5 eV) for the sulfur species of BPT and halogenated BPT as a function of irradiation time/dose. A saturation of the ratio can be interpreted as self-termination of the cross-linking of thiol substituted molecules [16]. Here, the raw data of the ratio is fitted by an unweighted exponential fit of the type *A*(1−exp(−*kx*)), implying that 2-I-BPT cross-links the fastest, followed by 2-Br-BPT, 2-Cl-BPT and finally BPT.

Figure 3 shows the XPS data of the sulfur S 2p region for SAMs from 2-Cl-BPT, 2-Br-BPT and 2-I-BPT. For reference, XPS data for the SAMs from native non-halogenated BPT are also shown. Similar to Figure 2, the XP spectra are recorded at nine different stages of irradiation within the time range from 0 to 120 minutes (0 to 72 mC/cm^2^). The raw data of four characteristic irradiation stages is plotted in Figure 3a.

**Figure 4:**
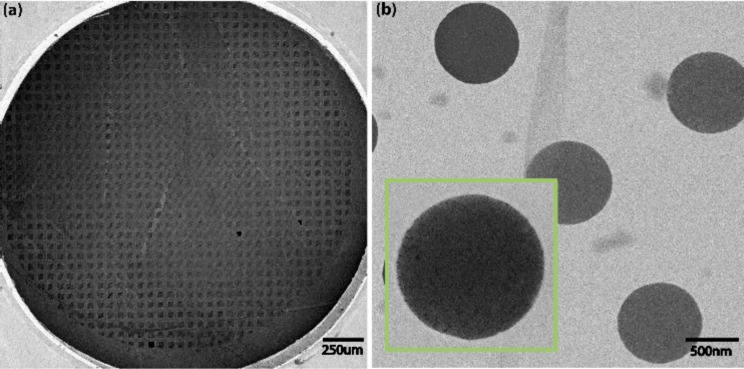
Helium ion microscope (HIM) image of a transferred carbon nanomembrane (CNM) made using 3 min (1.8 mC/cm^2^) of electron irradiation (50 eV) on a 2-I-BPT self-assembled monolayer. (a) 2500 × 2500 µm HIM image of the 2-I-BPT CNM transferred onto a TEM grid. (b) Magnification of (a) shows grid holes covered by a mechanically stable CNM with a distinct porosity (inset, green box).

The interpretation of the spectra of the halogenated as well as the conventional non-halogenated BPTs is based on the characteristic S 2p_3/2_/S 2p_1/2_ doublets for the S2p spectrum. The doublets were fitted presuming an energy separation of 1.2 eV [37] and the same FWHM was used for these spectra. The S 2p_3/2_ peak at BE = 162 eV is related to the thiolate anchor group at the end of the molecule attached to the Au(111) substrate in the native SAM. The other is related to the formation of new sulfur species, for example, disulfide, which appears with a S 2p_3/2_ peak at 163.5 eV and reflects the loss of thiol groups. A reduction of the 162 eV S 2p doublet and increase of the 163.5 eV, that is, a transformation of the relative ratio of the different S 2p doublet peaks, is visible for all four SAMs when comparing the first (0 min) and last (120 min) XP spectra of the molecules. This reduction can be taken as an indicator of the cross-linking process, as already mentioned above. Moreover, if the intensity ratio of the newly formed sulfur species (*I*_nSs_) and to the thiolate–Au peak (*I*_RS-Au_) is plotted vs the electron irradiation dose (or time), a saturation of the thiol group reconfiguration into the other sulfur species can be identified, implying completion of the cross-linking of the SAMs and the formation of CNMs [16]. Such an intensity ratio is plotted in Figure 3b showing the ratio *I*_nSs_/*I*_RS-Au_ for the sulfur species of BPT, 2-Cl-BPT, 2Br-BPT and 2-I-BPT along with an exponential fit applied to the data. Turchanin et al. observed a saturating electron dose for BPT between 40 to 60 mC/cm^2^, which is in accordance with the data (black curve) presented in Figure 3b [16]. Comparing this data with that obtained for 2-Cl-BPT (red curve) and 2-Br-BPT (green curve), a slight trend towards a more effective cross-linking of 2-Br-BPT seems to be visible, despite the noise present in the XPS raw data (Figure 3a). Nevertheless, the plots of both of these SAMs show saturation, that is, a self-termination of the cross-linking, between 12 and 24 mC/cm^2^ (20–40 min). In contrast, the data for 2-I-BPT reveals a very effective cross-linking, compared to the other halogenated and non-halogenated SAMs. A saturation of the intensity ratio plot already occurs between 1.8 and 3 mC/cm^2^ (1–3 min). This is more than 10 times faster than that observed for BPT and the other halogenated BPTs. Such finding is remarkable, considering that the 2-I-BPT precursor molecule is only halogenated at a single position in of the upper phenyl ring. However, it should be taken into account that these compounds are also subjects to different steric restrictions determining their relative orientation. For instance, the twist angle between the phenyl rings is a result of a compensation of the repulsion of the ortho hydrogens, or halogen atoms respectively, and the delocalized π-electrons of the neighboring phenyl ring on the other hand. This interaction leads to a twist angle of 45° for BPT 60° for 2-Cl-BPT, 63.8° for 2-Br-BPT, and 67° for 2-I-BPT [38–39], respectively. This may affect the reorganization of the molecules when forming SAMs and is also likely to affect the cross-linking efficiency [40]. Secondly, when a SAM is formed on a metallic surface, such as Au(111) for example, the work function of the substrate is changed, leading to an increase or a decrease of the secondary electron emission. In the same manner, self-assembled monolayers from different terminated molecules can lead to an unequal change of the surface work function [41] as well as the emission of secondary electrons. However, the specific role of the halogen substituent within the cross-linking process cannot be extracted from our experimental data and should be investigated by further experiments. As stated above, a direct verification of CNM formation may be achieved by transfer of an irradiated SAM to another substrate, thus proving its mechanical strength. In Figure 4, HIM images of transferred 2-I-BPT CNMs are presented. Figure 4a shows a mechanically stable CNM, covering the whole TEM grid, after an electron irradiation time of 3 min, corresponding to an electron irradiation dose of 1.8 mC/cm^2^. Some ruptures within the membrane are visible, which were probably caused during the transfer process onto the TEM grid or which could also be induced by the PMMA cleaning process within the critical point dryer. Figure 4b and its inset (green box) clearly reveal the formation of a freestanding membrane. This confirms the observation of a mechanically stable and easily transferable CNM from 2-I-BPT after 3 min of irradiation, documenting that a strongly lowered electron dose of 1.8 mC/cm^2^ is an unambiguous proof of the successful cross-linking of a SAM and respective CNM formation. Comparative attempts were also made to transfer an irradiated SAM made from BPT, 2-Cl-BPT and 2-Br-BPT to other substrates or TEM grids after a lowered electron exposure of up to 15 min (9 mC/cm^2^). These experiments suggested that these layers were not stable enough to survive the transfer process, thus it was not possible to image an intact carbon nanomembrane derived from these precursors neither by optical microscopy nor by HIM.

## Conclusion

The high DEA cross sections observed at 0 eV for 2-I-BP as compared to 2-Br-BP and 2-Cl-BP, led to the hypothesis that radical formation through effective DEA channels may be purposely used to enhance the cross-linking efficiency for the production of CNMs. This hypothesis was put to the test through comparison of the cross-linking efficiency upon electron irradiation of SAMs produced from 2-I-BPT as compared to 2-Br-BPT, 2-Cl-BPT and BPT. XPS was used to follow the effect of electron irradiation on the bonding in the halogenated biphenyl-thiols, while a direct test of the mechanical strength and optical microscopy was used for more direct confirmation of the cross-linking efficiency.

The normalized intensity in the XPS data was shown to decrease for iodine, bromine and chlorine, confirming that the dehalogenation of 2-I-BPT takes place much faster than for 2-Br-BPT and 2-Cl-BPT at the same current density.

Furthermore, the extent and rate at which new electron-induced sulphur species are formed is considerably faster for 2-I-BPT as compared to the other halogenated BPTs and the native BPT. Based on this analysis, the cross-linking was more than ten times faster for 2-I-BPT as compared to the other halogenated and conventional BPT at the same current density. The efficiency of cross-linking in the 2-I-BPT SAMs was confirmed by successful transfer of a 2-I-BPT-based CNM, generated by an electron radiation dose of only 1.8 mC/cm^2^, to a TEM grid. This clearly shows the mechanical stability achieved through cross-linking of 2-I-BPT even at an electron radiation dose as low as 1.8 mC/cm^2^. This is to be compared to 40–60 mC/cm^2^ [16] which was needed to efficiently cross-link native BPT to form CNMs of comparable mechanical strength.

The superior performance of 2-I-BPT compared to 2-Br-BPT, 2-Cl-BPT and the native BPT strongly indicates that this enhanced cross-linking efficiency is rooted in the significantly higher DEA cross section of 2-I-BPT. This in turn opens up the possibility to use high cross-sectional DEA processes to purposely enhance the cross-linking efficiency of SAMs for the production of CNMs. Moreover, the site selectivity of the DEA process also offers the potential to direct the cross-linking to specific molecular sites for production of functional CNMs.

## Experimental

The 2-halobiphenylthiols were synthesized by a palladium-catalyzed Kumada reaction of the respective 1,2-dihalobenzenes (1-bromo-2-chlorobenzene in case of 2-Cl-BPT) with the Grignard reagent of (4-bromophenyl)triisopropylsilylsulfide [42]. The removal of the protecting silyl group was performed with methanol/HCl under strict exclusion of air [43]. The resulting thiols were purified by repeated chromatography. Details on the preparation will be published elsewhere. It is worth mentioning that, in particular, 2-I-BPT is quite light sensitive.

Dissociative electron attachment and dissociative ionization experiments were conducted in a crossed beam apparatus that has been described in detail elsewhere [44]. We thus only give a brief description here. A monochromatic electron beam generated in a trochoidal electron monochromator is crossed with an effuse beam of the target molecules that enters the reaction zone through a capillary connected to the inlet system through a high-precision dosing valve. To record the DEA ion yield curves, the negative ion fragments formed are extracted into a quadrupole mass filter set to only allow transmission of a single, selected ion fragment and the electron energy is scanned through the energy range studied (here 0–10 eV). To record the DI mass spectra, the electron energy is fixed at 70 eV and the transmission of the quadrupole mass filter is scanned through the relevant mass range. The base pressure of the instrument is about 5 × 10^−8^ mbar and the acquisition pressure was maintained at about 5 × 10^−7^ mbar. The electron energy resolution was around ≈110 meV, determined by the FWHM of the well-documented SF_5_^−^ formation from SF_6_. The energy scale was calibrated with respect to the SF_6_^−^ formation from SF_6_ at 0 eV. In the current experiment, the inlet system was maintained at room temperature, but to avoid condensation on the electrical lens, the components and resulting charging the monochromator is maintained at 120 °C by means of halogen lamps. Consequently, there is a temperature gradient along the inlet capillary and we expect the target gas to be above room temperature. From previous experiments we expect the gas temperature to be between 40 and 60 °C when entering the reaction zone.

The CNMs were prepared by electron irradiation of self-assembled monolayers of halogenated BPT at UHV conditions, meaning a chamber pressure of approximately 1 × 10^−10^ mbar. The electron energy was set to 50 eV and the electron bombardment was performed in nine incremental steps of irradiation time between 1 and 120 minutes. The electron dose was calibrated by means of a mobile Faraday cup built for the sample stage of the analysis chamber. The electron current between the flood gun and the cup was measured for an array of lateral positions. As a result, one minute of electron irradiation at a beam energy of 50 eV corresponds to an electron dose of 0.6 mC/cm^2^ on the sample. SAMs were grown by immersing a commercially produced Au substrate with (a 300 nm Au(111) layer sputtered on mica) into a solution of the relevant biphenyl diluted in dimethylformamide (DMF). After 72 h at 300 K, a homogenous molecular layer was formed. The Au(111)/mica substrate was precleaned by ozone cleaning and ethanol rinsing. The XPS experiments were also performed under UHV conditions using the monochromatic X-ray source XM1000 and the SPHERA hemispheric analyzer from Omicron Nanotechnologies. The analyzer was operated in the constant analyzer energy (CAE) mode at varying energies of 20–50 eV, depending on the element under detection. Since 2-I-BPT is highly sensitive to light exposure, its preparation and the experiments were performed either in the dark or using a yellow-light environment. The HIM images were recorded using a Zeiss Orion Plus HIM at 34.7 kV and with a blanker current of 0.3 pA at secondary-electron-detection mode. For a better resolution in the HIM, the samples were transferred to a Quantifoil R0.6/1 TEM grid with a mean hole diameter of 0.6 µm and an average hole–hole distance of 1 µm. For the exfoliation of the CNM from the gold, PMMA was used as a coating for the nanomembrane. With the help of aqueous iodine, the PMMA–CNM stack was detached from the gold substrate. Subsequently, the PMMA was dissolved by acetone within a critical-point dryer.
